# Is musical ability related to second-language acquisition? A meta-analysis

**DOI:** 10.1098/rsos.241193

**Published:** 2025-01-15

**Authors:** Rachel M. Thompson, Lauren K. Salig, L. Robert Slevc

**Affiliations:** ^1^ Program in Neuroscience and Cognitive Science, University of Maryland, College Park, MD, USA

**Keywords:** second-language acquisition, meta-analysis, musical ability

## Abstract

In our multicultural and interconnected world, the ability to learn new languages is important. However, there are significant differences in how successfully adults can learn aspects of non-native languages. Given robust relationships between musical ability and native-language processing, musical ability might also contribute to successful second-language acquisition. However, while several studies have assessed this relationship in various ways, the consistency and robustness of the relationship between musical ability and second-language learning remains unclear. Thus, we synthesized 184 effects across 57 independent studies (*n* =3181) with a robust variance estimation multivariate meta-analysis, and we narratively summarized partial correlation effects across 12 studies. The available evidence suggests that musical ability is indeed positively related to second-language learning, even after factoring in publication bias revealed by the meta-analysis. Although future work with more diverse participant populations and methodologies is needed to further disentangle this relationship, it is apparent that individuals with better musical ability are generally more successful at second-language learning.

## 1. Introduction

Humans have an impressive ability to learn the sounds and structure of their native linguistic and musical systems. However, this impressive ability to learn is diminished later in life, at least for language: adults’ ability to achieve native-like proficiency in a second language is notoriously variable, especially in the domains of phonology and morphosyntax [1–3].^1^ While reaching native-like proficiency in a second language is not the end goal for all language learners, many may strive for native-like proficiency to avoid the social stigma associated with accents [6,7] and to improve communication with native speakers [8,9]. Of course, this goal is met with varying success. Thus, two questions emerge: why are some individuals more successful than others in learning a second language, and what characterizes successful second-language learners?

One idea, supported by a growing body of research, is that successful second-language acquisition is related to musical ability. This follows from broader links between musical and linguistic processing in the native language (for reviews, see [10,11]), including evidence that individual differences in grammatical ability and speech processing are related to musical ability (e.g. [12–15]). For example, musical ability predicts the distinctiveness of neural responses to closely related speech sounds [16], speech discrimination performance [17], and sensitivity to prosody [13,18]. One framework to understand these types of music–language relationships is [19] OPERA hypothesis, according to which musical training promotes structural and functional changes in important auditory processing networks (*cf* [20]). Alternatively, these relationships might not reflect effects of musical training, but rather exist because music and language both rely on similar underlying abilities (see [21,22]). In either case, findings of associations between musical and native-language abilities are often taken as indication for deep, underlying relationships between music and language.

Although the specific reasons for music and language relationships are debated, it is relatively uncontroversial that musical and linguistic abilities are correlated. Indeed, recent meta-analyses have found evidence for relatively robust relationships between musical ability and native-language speech processing [23] and reading ability [12]. It is less clear, however, if this relationship extends to *second-language* learning. Some evidence suggests musical ability and musical training predict aspects of second-language proficiency, including perception and production of second-language phonology [24], perception of second-language prosody (see [13] for a meta-analysis), lexical tone discrimination [25,26], and speech segmentation [27,28]. However, other evidence suggests that musical ability or training is unrelated to second-language learning (e.g. [29,30]). For example, some studies have found no clear link between musical ability and vowel learning [31], phonemic discrimination [32], and self-rated second-language abilities [33]. These varied results suggest a need for a formal meta-analytic assessment of the relationship between second-language abilities and musical experience/ability.

## 2. The present study

In light of these mixed results, we aimed to systematically assess existing findings on music and second-language relationships by conducting a meta-analysis on the relationship that musical ability (which here refers to performance on assessments of the perception or production of musical notes, melodies, or rhythms) and musical training (which here refers to the duration of prior musical experience) have with second-language learning. We also investigated several factors that might moderate such a relationship, including the type of musical measure used (e.g. ability assessment or self-reported training), the type of second-language learning assessed (e.g. lexical tones, syntax, or segmental phonology), and learner characteristics (e.g. age or type of native language). As noted above, there are theoretical reasons to expect a link between music and second-language learning (e.g. [19]), and two narrative reviews of the relevant literature have concluded that there is, indeed, such a link (albeit a decade ago [34,35]). However, there has not yet, to our knowledge, been a systematic meta-analysis of music and second-language relationships. Such a meta-analysis offers several benefits over narrative reviews, such as allowing for the quantification of effect sizes, assessment of potential moderating effects, and assessment of the quality of the literature (i.e. in terms of publication bias). This approach thus can offer a better understanding of the depth, breadth, and nuances of music–second-language relationships that we hope will help illuminate future research avenues.

## 3. Method

### 3.1. Search strategy

**Figure 1.**
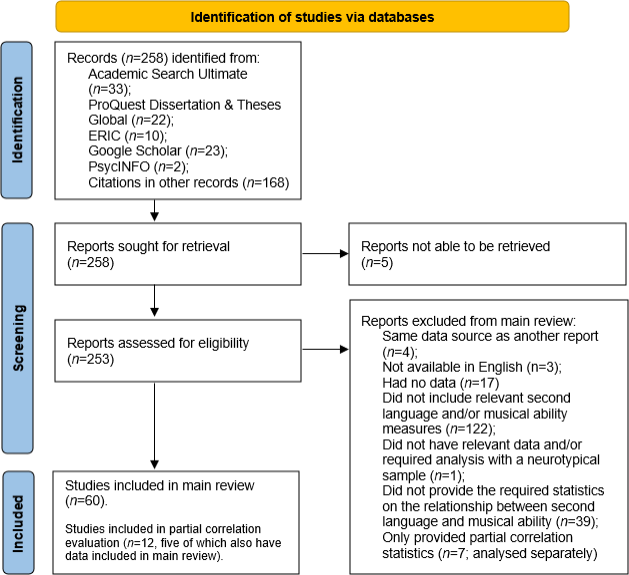
Flow chart of literature search and study selection. Note: the PRISMA diagram outlines the various steps of the literature search for study inclusion and exclusion. Reasons for exclusion are indicated at each step.

The current meta-analysis was carried out in compliance with the Preferred Reporting Items for Systematic Review and Meta-Analyses (PRISMA [36]). We performed a literature search across six databases, searched reference lists within relevant articles for studies, and consulted five review articles to select articles to include in analyses. Our search syntax for databases was: (second language OR foreign language OR bilingual) AND (musical ability OR musical experience OR musical aptitude OR musical training OR musicality), entered as a Boolean phrase. In total, we found 258 potentially relevant articles, of which 60 met our inclusion criteria. These 60 studies included 189 effect sizes and a total of 3462 participants (see figure 1 for the literature review process). The data and analysis scripts are available on the Open Science Framework (OSF): https://osf.io/4yzpq/.

### 3.2. Inclusion criteria

To be in the final analyses, studies must have met the following inclusion criteria:

- must be a published article or unpublished thesis/dissertation in English;
- must include a clear behavioural measure of musical aptitude, experience, or training. This could be a continuous measure based on scores from a test (e.g. Advanced Measures of Musical Audition [37]), participants’ self-report of musical training/experience, or groups defined from scores on a musical test.^2^ Alternatively, experimenters could have provided musical training to participants as a form of a musical intervention;
- must include a clear behavioural measure of second-language learning/ability. This could include measures of second-language perception (e.g. discriminating between phonemes or lexical tones) and/or second-language production (e.g. repeating phonemes);
- must assess participants without identified neurological problems, learning impairments, or language impairments. If the sample included non-neurotypical participants, the study must also provide a relevant analysis including only neurotypical participants; and
- must quantify the relationship between musical and second-language measures or predict second-language learning from musical measures via a Pearson correlation coefficient (*r*) or another statistic that could be transformed into a correlation coefficient (e.g. *t*, Cohen’s *d* or *F*; see §3.3). Studies that reported partial correlations (such as findings from a multiple regression or partial correlation analysis) were excluded from the main analyses, but several such studies are summarized narratively below (see §4.4).

### 3.3. Converting among effect sizes

Although most reported effect sizes were correlations, it was necessary to convert and transform effects that were reported as standard mean differences or with other effect size metrics. We used two web-based calculators to calculate and convert effect sizes: Campbell collaboration’s online calculator [38] to calculate effect sizes for standard mean differences and escal [39] to convert effect sizes to Pearson’s *r*. We then converted all values to *z*-scores via the Fisher *z*-transformation.

### 3.4. Moderator variables

A common challenge with meta-analyses is that studies on the same topic vary considerably. One way to address this issue is to consider specific dimensions on which studies systematically differ as potential moderators of an effect. As such, we identified (based on past work or on plausibility) six factors that seemed likely to relate to the strength of the observed music and second-language relationship:

- age (children or adults): learning the novel sounds and structures of a second language seems to become more difficult as people age [1,40], suggesting that musical ability—second-language relationships might also differ by age. In the studies examined here, participants’ age varied from 5 to 69 years; however, the distribution was relatively bimodal: studies generally included either relatively young (5−9 years old) or adult (18−69 years old) participants. We thus classified participant groups that were under 15 years old as *children* and groups over 16 years old as *adults*;
- musical measure (ability, training or intervention): there is considerable variability in conceptualization and assessment of musical ability and musical training across studies: some researchers measure perceptual skills with a standardized test, while others assess self-reports of musician status or use a musical intervention. This may introduce variability in the strength and direction of any observed relationship between music and second-language ability. As such, we examined how the operationalization of musical ability related to music–second-language learning effect sizes. Studies that assessed musical ability with a standardized test (e.g. the Profile of Music Perception Skills, or PROMS [41]) or an experimenter-made test were classified as musical *ability* studies. Studies that divided participants into musicians and non-musicians, asked for participants’ self-reported musicianship, or used years of musical experience as a continuous variable were classified as musical *training* studies. Studies that provided musical training as an intervention (e.g. music lessons versus a control group) were classified as *intervention* studies. However, the very limited number of intervention studies forced this to be treated as a two-level factor (ability/training) for the moderator analyses;
- language measure (phonology or non-phonology): as noted above, work on second-language acquisition in adult learners has suggested particular variability in phonological abilities and morphosyntactic abilities (compared to lexical knowledge; e.g. [1]), and so any relationship with musical ability may differ depending on which aspect of the second language is assessed. Note, however, that most of the literature has focused on second-language phonology (80% of the effects assessed here); thus, we classified effects as either measuring aspects of participants’ abilities in second-language *phonology* (80% of effects) or in non-phonological aspects of second-language abilities;
- language modality (perception or production): the perception of a second language (e.g. discriminating between phonemes) and production of a second language (e.g. repeating/imitating phonemes) may rely on different abilities. For example, an individual may accurately discriminate between second-language sounds but be unable to produce those sounds in a native-like way. As such, we used a two-level moderator that described if the second-language measure was about language perception or language production. Studies that assessed participants’ ability to discriminate between phonemes or discriminate/categorize aspects of second language were classified as *perception* studies and work that assessed participants’ abilities to imitate or produce aspects of a second language were classified as *production* studies;
- tone/non-tone language (tone or non-tone): some work has suggested specific links between musical (pitch) abilities and the learning of languages that use pitch contrastively (i.e. tone languages; e.g. [42]). Thus, we classified effects as involving a second language (or languages) that was a *tone* language (e.g. Mandarin) or a *non-tone* language (e.g. English). Note that this distinction is only relevant for studies measuring second-language phonology:
  - we identified five studies (with 28 effect sizes) that involved ‘pitch accent’ languages, which use pitch contrastively but to a lesser degree than tone languages (specifically Swedish, Norwegian, and Japanese: [43,44,45,46,47]). This sample was too small to include as a moderator and, because some have argued that pitch accent may not be a coherent category (e.g. [48]), we grouped these with the *tone* languages; and
  - we also coded whether participants’ *first* language was a tone or non-tone language; however, we identified only two studies that indicated that participants’ first language was a tone language [49,50]. This sample was also too small to analyse as a moderator;
- language learning outcome (proficiency, novel or learning success): studies differed in the type of language outcome they investigated. Some examined mastery of the second language, typically based on standardized language proficiency tests; some assessed participants’ initial language learning abilities by focussing on the discrimination and/or repetition of novel phonemes; and some measured participants’ learning over somewhat longer timeframes (i.e. improvement from pre to post-test, following an intervening language class or experimental teaching intervention). Such effects were classified as assessing *proficiency*, *novel learning* and *learning success* outcomes, respectively.

### 3.5. Screening and data extraction

We conducted abstract and full-text screening procedures. First, we curated a list of articles that could match our inclusion criteria from our initial search syntax. Then, two co-authors screened abstracts and full texts to determine if they matched our inclusion criteria. If articles failed one or more inclusion criteria, they were excluded from the subsequent review processes after being checked by another author to ensure accuracy. All coding and exclusion disagreements were resolved by discussion.

We extracted study-level information such as participant age (children or adults), number of participants, participants’ native language, the second language assessed, and publication status (published or unpublished studies). We extracted characteristics on musical ability—specifically how musical ability was operationalized and/or how researchers defined musicians and non-musicians (if reported). We extracted characteristics on language measures—specifically whether they were perception or production measures and whether they focused on second-language phonology or lexical/syntax measures. Finally, we extracted any relevant effect sizes that quantified a relationship between musical ability and a second-language outcome. If experiments included subcomponents of musical batteries (e.g. pitch perception and rhythm perception for PROMS) and total scores, only subcomponents were included in final analyses to avoid double counting.

## 4. Results and discussion

### 4.1. Main analyses

**Figure 2.**
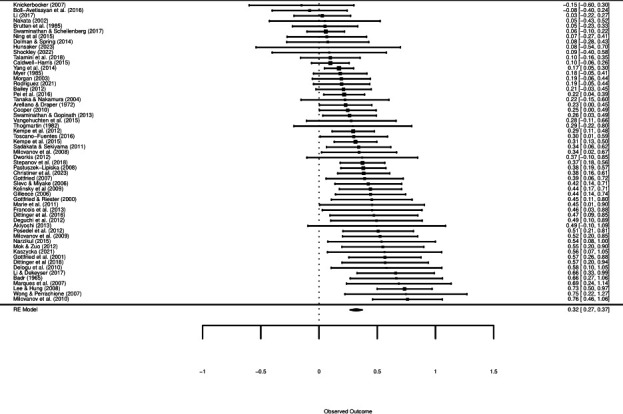
Forest plot of musical ability/training on second-language learning. Note: for illustrative purposes, we plotted each experiment’s average effect size (Fisher’s *z*-score) following guidelines from Hedges *et al*. [54] and Linck *et al*. [55] and ordered them from smallest to largest. Each dot differs in size based on study weight; larger dots indicate larger sample sizes while smaller dots indicate smaller sample sizes. Confidence intervals are given in brackets.

To account for multiple correlated effects, we performed a robust variance estimation multivariate meta-analysis using the *robumeta* package (version 2.0 [51]) in R (version 4.1.2 [52]) with random effects of study where ‘study’ refers to results from one experiment. Each effect size was reported as a Fisher’s *z*-score. Outlier analyses assessed each effect size’s studentized residuals and Cook’s distance, which identified five potential outliers across three studies ([25,26,53]). These studies contributed implausible effect sizes (Fisher’s *z* > 1) with large residuals. As such, these outliers were removed, which reduced our sample to 57 studies and 184 effect sizes. Then, we ran analyses with and without these effect sizes and observed the overall meta-analytic effect decrease from *z* = 0.36 to *z* = 0.32. While the overall meta-analysis showed a positive, significant relationship between musical ability and second-language learning, this effect was noticeably inflated (and not created) by the presence of implausible outliers. See figure 2 for a corresponding forest plot (for visualization purposes, this plot shows data aggregated by study; a plot including all 184 effect sizes can be found at https://osf.io/4yzpq/). This analysis shows that past work on musical ability and training (broadly defined) and second-language learning does reveal a positive relationship overall (*cf* [34,35]).

However, meta-analytic estimates can be strongly influenced by publication bias. For example, if studies with non-significant or negative effects are less likely to be published (the ‘file-drawer problem’), this leads to an over-representation of larger effect sizes which can lead to an overestimation of, and potentially incorrect conclusions about, the meta-analytic effect [56,57]. Therefore, the next section details how we assessed, corrected and adjusted for possible publication bias.

### 4.2. Publication bias

We relied on three methods to detect and quantify publication bias in our meta-analysis. First, we assessed publication status as a moderator, given that unpublished findings (e.g. unpublished theses and dissertations) are presumably less affected by publication bias than published work. Specifically, we compared 68 effect sizes from the 15 unpublished dissertations/theses identified in our literature search to the effect sizes from the remaining 42 published papers (116 effect sizes); these effects did not differ significantly (*z* = 0.27 and *z* = 0.34 for unpublished and published studies respectively; *t*_20.6_ = 1.23, *p* = 0.23).

**Figure 3.**
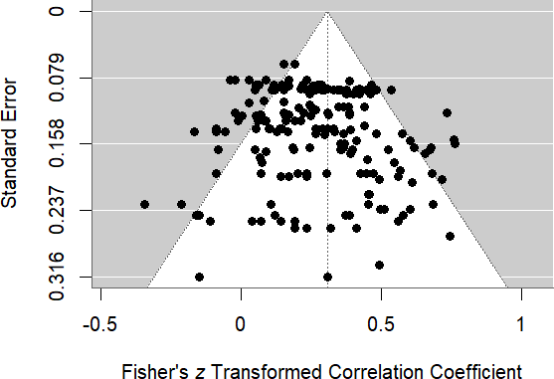
Funnel plot of all effect sizes. Note: all effect sizes (black dots) plotted against their standard errors. In the absence of publication bias, points should be symmetrically distributed around the meta-analytic effect (dotted line) with variability increasing as precision (as indexed by standard errors) decreases.

Second, we created a funnel plot (figure 3) which plots each effect size against its standard error and illustrates the overall meta-analytic effect with a dotted line. In the absence of publication bias, variation around the overall meta-analytic estimate should: (i) be symmetrically distributed around the meta-analytic effect; and (ii) decrease with increasing precision (indexed as decreasing standard error), thus generally occupying the ‘funnel’ shape in white. Visual inspection of the funnel plot suggested some degree of asymmetry, such that the more precise estimates (at the top) appear to be shifted left from the overall meta-analytic effect, and some imprecise estimates (at the bottom) are positive and relatively large. Both of these patterns would be consistent with publication bias. To complement this visual inspection of the funnel plot, we assessed for asymmetry with an Egger’s regression test [58], which also indicated some funnel plot asymmetry (*t* = 2.26, *p* < 0.03, 95% confidence interval [0.13 0.30]). However, note that an asymmetric funnel plot is not necessarily evidence of publication bias (e.g. [59]), and even if publication bias exists, this does not necessarily indicate a non-existent effect, but rather might reflect an inflated estimate of an effect that does, in fact, exist.

Third, we more formally quantified and adjusted for the extent of publication bias by relying on the *precision-effect test* and *precision-effect estimate with standard errors* (PET-PEESE [60]). This approach adjusts the relationship between effect sizes and standard errors with two steps: the PET model first predicts effect sizes with their standard errors with a weighted linear regression, while the PEESE model predicts the effect sizes with squared standard errors and then adjusts the overall meta-analytic effect for publication bias [61]. If the PET model is statistically significant, the PEESE test can be interpreted as the meta-analytic effect adjusted for publication bias. The PET test (table 1) was indeed statistically significant, and accounting for this publication bias yielded an adjusted effect size of *z* = 0.25, which is notably smaller than the uncorrected meta-analytic effect of *z* = 0.32 but still indicates a statistically significant positive relationship between musical ability and second-language learning.

**Table 1.** PET-PEESE Mean estimates. (Note: tests conducted following guidelines from Roman-Caballero *et al.* [62] and Bartoš *et al*. [61].)

						95% confidence interval	
	estimate	s.e.	*t*	d.f.	*p*	lower	upper
PET	0.15	0.07	2.26	22.8	0.03	0.01	0.30
PEESE	0.25	0.04	5.94	27.2	<0.001	0.17	0.34

Taken together, these analyses suggest that publication bias is indeed an issue present within this literature, but it appears to inflate, rather than create, the measured relationship between musical ability and second-language learning. Given this evidence that there is indeed a (modest) relationship between musical and second-language abilities, we assessed how this relationship varies across various potential moderators.

### 4.3. Moderator analyses

Although the effects making up this meta-analysis all address some aspect(s) of musical and second-language abilities, they come from studies that vary considerably in terms of both participant populations and methodologies. Therefore, we ran six robust multivariate meta-regressions to assess moderators of interest [51]. Table 2 contains a summary of all moderator findings with relevant statistics.

**Table 2.** Moderators with analyses (not corrected for multiple comparisons).

moderator	no. of effect sizes	% of sample	*z*	*t*_d.f._	*p*
age				*t*_23.7_ = −0.32	0.75
adults	121	67	0.35		
children	63	33	0.31		
musical measure				*t*_45_ = 0.72^a^	0.47
musical ability	134	70	0.30		
musical training (self-report)	47	28	0.34		
musical intervention^b^	3	2	0.58		
language measure				*t*_12.4_ = 1.52	0.15
phonology	148	80	0.29		
syntax/semantics	36	19	0.17		
language modality^c^				*t*_35.5_ = −0.63	0.53
perception	86	65	0.36		
production	62	35	0.30		
tone language^c^				*t*_23.3_ = 1.92	0.07
tone	21	17	0.44		
non-tone	127	83	0.28		
language learning outcome					
novel	104	56	0.35		
learning success	21	12	0.38		
proficiency	59	32	0.22		

^a^ This test does not include the four intervention effect sizes because of limited sample size, the sample for this comparison is reduced to 55 studies.

^b^ These four intervention effect sizes are not included in the moderator analysis but are included in the overall meta-analysis.

^c^ This test includes the effect sizes involving aspects of second-language phonology.

#### 4.3.1. Age (children or adults)

Given discussions of critical and sensitive periods for second-language acquisition (see [1] for discussion), we investigated if relationships between musical ability and second-language learning differed for adults (here: aged 16 and above) and children (aged 15 and below). Descriptively, children showed smaller effects (*z* = 0.31) than adults (*z* = 0.33); however, this comparison was not statically significant *t*_23.7_ = −0.32, *p* = 0.75. Therefore, the relationship between musical ability and second-language learning does not appear to change across age of learning in this sample, although the lack of a moderator effect may be driven by the need to collapse age into a dichotomous category for this analysis rather than investigating age as a continuous variable. Nevertheless, this null result was somewhat surprising given the well-documented advantage in second-language ability for younger learners (e.g. [40,63]), which indicates that younger learners should have less variability in second-language outcomes overall (and thus less variability to be predicted by individual difference factors like musical ability). For example, one might have predicted that adult learners with relatively poor language learning abilities could rely on their musical abilities as a kind of compensatory mechanism, in which case music–second-language relationships should be stronger in adults compared to younger learners (*cf* [64]). In the case of music practice and age-related cognitive decline); however, we see no evidence to support this idea.

#### 4.3.2. Musical measure (ability, training, or intervention)

Typical investigations of music and second-language relationships measure musical ability based on participants’ ability to discriminate between minimal musical pairs (e.g. [41,65]), or based on participants’ self-reported status as a (non)musician or total years of musical training/experience. More rarely, investigations employ a musical intervention, where listeners are given musical training and are compared to a control group. We thus assessed whether music and second-language relationships varied by the type of musical measure. Because there were very few musical intervention effect sizes in our sample (*n* = 3) compared to musical ability (*n* = 134) and musical training studies (*n* = 47), our moderator analysis only statistically compared effect sizes for musical ability and musical training studies. There was no significant difference between the effect size in studies measuring musical ability (*z* = 0.30) and those measuring musical training *z* = 0.34; *t*_45_ = 0.72, *p* = 0.48. Note that, descriptively, the four intervention studies did show a notably larger effect: *z* = 0.58.^3^ Of course, performance on musical ability tasks is probably correlated with musical experience [66,67], so different relationships of these factors on second-language acquisition may be difficult to observe; nevertheless, these data suggest that second-language learning is similarly related to measures of musical abilities and musical experience.

#### 4.3.3. Language measure (phonology or syntax/semantics)

Sound is perhaps the most obvious connection between musical ability and second-language learning, and indeed a majority (80%) of the effect sizes in our sample focus on speech processing (i.e. perception or production of second-language sounds/phonology). However, music and language may be related across multiple levels of representation [10,11], and some work in our sample assessed other aspects of second-language abilities (e.g. lexical or syntactic). This moderator effect was not statistically significant, though effects involving second-language phonology were larger (*z* = 0.34) than effects involving other aspects of second-language abilities (*z* = 0.25; *t*_12.41_ = 1.52, *p* = 0.15). It is likely, however, that this moderator was impacted by the removal of outliers.

#### 4.3.4. Language modality (perception or production)

Accurate speech sound discrimination does not necessarily equate to native-like production [68], suggesting that musical relationships with perceptual and productive aspects of a second language might differ. Therefore, we assessed if there were differences between studies assessing second-language perception and those assessing second-language production. We observed no significant difference in this moderating effect: musical ability was similarly related to perceptual (*z* = 0.35) and productive (*z* = 0.31) aspects of second-language learning (*t*_35.5_ = −0.63, *p* = 0.53).

#### 4.3.5. Language tone type (tone or non-tone)

Pitch plays a lexically contrastive role in tone languages which poses difficulties for non-native listeners and speakers [26,42]. Because pitch plays a central role in musical processing, it is plausible that musical ability and training may relate especially to the ability to perceive, produce, and categorize lexical tone. Therefore, we compared studies of musical relationships with the learning of tone and non-tone second languages. Typically, studies on tone language learning involve categorizing lexical tones (e.g. [69]), discriminating between tonal contrasts (e.g. [25]), or producing various lexical tones (e.g. [70,71]). Studies investigating tonal aspects of a second language did not yield significantly a larger effect size (*z* = 0.44) than studies measuring learning in non-tonal aspects of a second language (*z* = 0.30; *t*_23.3_ = 1.92, *p* = 0.07).

#### 4.3.6. Language learning outcome (novel, learning success, proficiency)

The studies examined here operationalized second-language ability in multiple ways. Some studies assessed initial abilities/aptitudes with aspects of an unfamiliar second language, for example focusing on the discrimination or repetition of novel second-language phonemes (here, *novel* studies). Other studies assessed the effectiveness of second-language learning over a short duration, for example by comparing scores on some measure(s) before and after a short second-language learning intervention (here, *learning success* studies). Also, yet other studies assess proficiency in an already-learned second language, typically via performance on some kind of standardized test (here, *proficiency* studies). Each of these learning assessments could plausibly have quite different relationships with musical ability; therefore, we performed a moderator analysis that assessed how the relationship between musical ability and second-language learning varied based on different second-language outcomes (novel, learning success, or proficiency). Indeed, effect sizes differed significantly between these three groups. Numerically, studies measuring short-term learning yielded the largest effect size overall (*z* = 0.43) compared to studies examining novel learning (*z* = 0.36) and studies of eventual proficiency (*z* = 0.22). Follow-up robust variance estimations meta-analyses comparing these groups revealed no significant differences between learning success and novel studies (*t*_11.1_ = 0.32, *p* = 0.75). Instead, proficiency studies had significantly smaller effects than novel studies (t_22.6_ = −2.28, *p* = 0.03); this suggests that musical abilities are more strongly related to the immediate (or relatively short-term) perception/production of second-language sounds rather than overall proficiency in the second language.

#### 4.3.7. Summary of moderating effects

The relationship between musical ability and second-language ability only varied across immediate perception/production of short-term learning compared to assessed proficiency in a second language. Instead, relationships between musical ability did not vary across phonology and lexical/syntactic aspects of second-language learning, neither for tone compared to non-tone languages, and for immediate perception/production and short-term learning compared to assessed proficiency in a second language. Moreover, relationships between musical ability and second-language learning did not appear to vary as a function of age (in children versus adult learners), the modality of the language measure (perception versus production), or the type of musical measure (ability versus training).

Of course, many other factors might moderate these observed music and second-language relationships, and many of these cannot be easily investigated in group comparisons. However, it is possible to measure and statistically control for some of these individual differences. These sorts of partial correlation and multiple regression studies can be problematic to include in meta-analyses (e.g. [72]), so, in the following section, we detail a narrative synthesis of studies that have controlled for various factors to assess if musical ability can account for unique variance in second-language learning.

### 4.4. Partial effects (narrative synthesis)

The analyses above focus on studies assessing zero-order correlations between musical and second-language ability. Some studies, however, have investigated this relationship while statistically controlling for other potentially confounding individual differences such as working memory, prior language experience and intelligence, with the aim of assessing whether musical ability can explain *unique* variance in second-language learning. These types of effects were not included in the meta-analysis above because such partial correlations can have different mathematical properties than bivariate correlations and because correlations adjusted for different covariates are estimating different population parameters and so have different interpretations [72,73]. In addition, if covariates suffer from measurement error or represent an inappropriate causal structure, statistical control can introduce considerable bias [74,75].^4^

Nevertheless, these partial effect sizes certainly play an important role in our understanding of music and second-language relationships, and so here we summarize existing studies of musical ability and second-language learning that reported effects after controlling for various other factors. We followed the same inclusion criteria as our main analysis (see §3) which yielded 12 independent articles reporting 27 partial effect sizes from 643 participants (note that zero-order correlations from some of these same datasets are included in the analysis above). The average sample size in these studies was larger than our main analysis (average *n* = 72.8 in these 12 studies compared to an average *n* = 57.7 for the 60 studies above), and these studies primarily assessed musical and second-language learning abilities in adult participants.

Most commonly, studies have controlled for individual differences in prior language experience (e.g. exposure to the second language or starting second-language proficiency). Researchers have also controlled for individual differences in working and/or short-term memory [24,77,78], intelligence [33,78,79], motivation [79] and experimental context (perception or production training [71]). Table 3 lists each study, their second-language and music measures, what they controlled for, and their corresponding partial effect sizes.

**Table 3.** Summary of partial effects. (Asterisk (*) indicates significance at *p* < 0.05 level.)

study	*n*	Second-language measure(s) (outcome)	age (adult/child)	music measure(s) (predictor)	partialled out	partial effect size
Badr [79]	28	French vocabulary	child	pitch perception	intelligence	*r* = 0.60*
Badr [79]	28	French vocabulary	child	pitch perception	motivation	*r* = 0.61*
Badr [79]	28	French comprehension	child	pitch perception	intelligence	*r* = 0.55*
Badr [79]	28	French comprehension	child	pitch perception	motivation	*r* = 0.56*
Bowles *et al*. [77]	160	Mandarin pseudoword accuracy	adult	musical training	short-term memory	*β* = 0.01*
Cooper & Wang [26]	54	post-training Cantonese tone identification	adult	musical training	pre-training Cantonese tone identification	*r = 0*.39
Gotz *et al*.[80]	36	Thai speech perception	adult	musical training	rhythm perception	*β* = 0.06*
Gotz *et al*.[80]	36	Thai speech production	adult	musical training	rhythm perception	*β* = 0.25*
Jacobsen [81] — male participants	10	Japanese proficiency	adult	musical training	prior language exposure/experience, arrival age	*β* = 0.02
Jacobsen [81] — female participants	10	Japanese proficiency	adult	musical training	prior language exposure/experience, arrival age	*β* = −0.03
Jekiel & Malarski [82]	50	English accent production and phonological awareness	adult	musical training	starting English proficiency, pitch/rhythm perception, gender	*β* = −0.002*
Li & DeKeyser [71]	38	Mandarin tone production	adult	pitch perception	treatment group (perception/production training)	*η*_p_^2^ = 0.13^a^*
Li & DeKeyser [71]	38	Mandarin tone production	adult	pitch perception	treatment group (perception/production training)	*η*_p_^2^ = 0.05
Mokari & Werner [31]	38	English phoneme perception	adult	pitch perception	starting second-language proficiency	*r* = 0.29
Mokari & Werner [31]	38	English phoneme production	adult	pitch perception	starting second-language proficiency	*r* = 0.18
Schellenberg *et al*. [33]	154	self-rated English proficiency	adult	pitch perception	gender, education, cognitive ability	*r* = 0.06
Schellenberg *et al*. [33]	154	self-rated English proficiency	adult	rhythm perception	gender, education, cognitive ability	*r* = 0.02
Schellenberg *et al*. [33]	154	self-rated English proficiency	adult	musical training	gender, education, cognitive ability	*r* = 0.05
Schellenberg *et al*. [33]	154	self-rated English proficiency	adult	singing ability	gender, education, cognitive ability	*r* = 0.06
Slevc & Miyake [24]	50	English receptive phonology	adult	aggregate of multiple tasks	age of arrival, length of residence in US, language use/exposure, phonological short-term memory	*β* = 0.37*
Slevc & Miyake [24]	50	English productive phonology	adult	aggregate of multiple tasks	age of arrival, length of residence in US, language use/exposure, phonological short-term memory	*β* = 0.30*
Slevc & Miyake [24]	50	English grammaticality judgements	adult	aggregate of multiple tasks	age of arrival, length of residence in US, language use/exposure, phonological short-term memory, language use/exposure, phonological short-term memory	*β* = 0.13
Slevc & Miyake [24]	50	English vocabulary	adult	aggregate of multiple tasks	age of arrival, length of residence in US, language use/exposure, phonological short-term memory	*β* = 0.04
Swaminathan & Schellenberg [78]	151	Zulu phoneme identification	adult	rhythm perception	short-term memory working memory	*r* = 0.25*
Swaminathan & Schellenberg [78]	151	Zulu phoneme identification	adult	rhythm perception	short-term memory, working memory, music training, nonverbal intelligence	*r* = 0.26*
Tabori [83]	95	Mandarin tone Identification	adult	musical training	native language_^b^_	2.47 Odds ratio*

^a^ This value reflects a combined score from three tasks.

^b^ Participants were either Spanish–English bilinguals, Bantu–English bilinguals or Vietnamese–English bilinguals.

In contrast to the clear relationship indicated by the overall meta-analytic results reported above, just over half (53.8%) of these partial effect sizes were statistically significant after controlling for other factors. Nearly all of these positive relationships were observed for studies measuring second-language phonology, although one study [79] found a significant partial correlation between musical ability and second-language vocabulary and comprehension. There was no obvious pattern in the factors controlled for in studies that yielded significant versus non-significant partial music–language effects. Of course, more than half of these partial effects were significant and statistical control can artificially inflate both type I and type II errors (e.g. [84,85]), so it is not yet clear what to conclude from this small body of work. Nevertheless, this result does underscore the importance of considering other, possibly confounding, factors.

## 5. General discussion and conclusion

Adult second-language learning success exhibits incredible variability: some struggle to discriminate between and produce non-native sounds at their desired level, while others reach native-like proficiency [1]. There are surely many individual differences that contribute to this variability (e.g. [86]), one of which may be individual differences in musical ability. This is sensible given relationships between musical ability/training and native-language speech processing (e.g. [12,15,23]). However, while many studies have investigated links between musical ability/training and second-language learning, the robustness and consistency of such a relationship has remained unclear (e.g. [31,33]). Here, we rely on meta-analytic methods to better understand if, and how strongly, musical ability is related to successful second-language learning.

We synthesized 184 effect sizes across 57 independent articles with a robust variance estimation multivariate meta-analysis. The overall size of the meta-analytic effect was small to moderate according to Cohen’s (1992)[87] standards (*z* = 0.32); furthermore, this effect appears to be inflated owing to publication bias. After bias correction methods (PET-PEESE), the meta-analytic effect remained statistically significant, but considerably smaller (*z* = 0.25): a small effect per Cohen’s standards (1992)[87]. Thus, we conclude that the effect reflected in the literature is inflated, but not created. This effect size fits with similarly small meta-analytic effects observed for other types of music–language relationships (e.g. [12,23]). While the overall effect at its adjusted size is small, it nevertheless supports a long-speculated relationship between musical ability and second-language learning and highlights how connections between musical and language processing extend beyond the native language ([19,34,88,89]; among others). This relationship may also have pedagogical implications for music teaching and second-language learning; for example, these findings fit well with the idea that including music in classrooms or participating in musical activities could assist in the perception and production of non-native sounds (see [90] for a review of music in classroom use). In summary, the present meta-analysis provides evidence that musical ability is indeed positively, albeit modestly, related to second-language learning outcomes across a variety of participant populations and methodologies.

Despite the depth and breadth of the literature examined, the overwhelming percentage of effect sizes assessed here were correlational; thus, this meta-analysis cannot confirm a causal relationship or offer support for ‘transfer’ between music and second-language learning. However, note that the three effect sizes from the two intervention studies included [42,91] found larger effects than musical ability and musical training studies, offering suggestive evidence for some type of transfer. This is plausible given that both music and language are mediated through experience and give rise to training-specific plasticity, probably specific to auditory learning [92,93]. Regardless, the observed relationship is interesting even if it does not reflect a causal effect. Music and language may be the best demonstrations of our ability to perceive and process complex auditory sequences [19]. Better understanding music and language relationships could help us understand the underlying mechanisms that give rise to our ability to integrate and extract meaning from sound.

Given the variability in measures and methods across the included studies, we assessed how musical ability and second-language relationships varied across six moderators. Only one moderator was statistically significant, studies measuring learning success (i.e. change from pre- to post-test) and novel second-language learning yielded larger effect sizes than studies of achieved second-language proficiency. This suggests that music and second-language relationships may be limited to (or at least stronger for) the initial perceptive and productive aspects of second-language learning rather than ultimate attainment of second-language proficiency. Such a notion is probably unsurprising given the many factors that influence real-world second-language learning that are not captured with these experiments (e.g. motivation [94]). One possibility is that more musical individuals might have an advantage during early stages of second-language learning owing to more accurate perception and production of second-language sounds—probably scaffolded by better auditory processing. However, these differences appear to dissipate over longer periods of time, resulting in smaller relationships with eventual second-language proficiency. In other words, musical ability might predict the ‘efficiency’ of second-language learning, but be less predictive of whether or not learners ultimately achieve high levels of second-language proficiency (e.g. [95]).

Interestingly, the relationships between musical ability/training and second-language learning did not appear to vary as a function of age (i.e. were no different for studies of adults and of children learners), second-language phonology and syntax, language modality (studies of language perception versuslanguage production), tonal aspects of second language versus non-tonal aspects of second language or assessment of musical training/experience versus musical ability. While interpretation of such null effects should be approached with caution, it is possible that musical ability relates similarly to both language perception and language production given that perceiving and producing language and music requires fine-grained auditory precision and motor control [19,88]. Similarly, it is likely that musical ability relates similarly across language phonology and syntax given the connection between music and sound processing (e.g. [11]) and relationships between musical ability and grammatical ability (e.g. [12]). Given that there were no observed differences between studies assessing musical training/experience and studies assessing musical ability (via performance on some kind of musical task), it may be that this relationship is not (solely) experience-based (and note that while musical ability and musical training are, of course, related, they are also dissociable, e.g. [41,96]).

Although not part of the meta-analysis itself, we also summarize several studies estimating musical ability’s unique contribution to second-language learning (i.e. partial correlation effect size estimates). This analysis was somewhat consistent with the meta-analytic results: many (but far from all) of these studies found statistically significant positive relationships even after controlling for various other non-musical factors. These included measures of various cognitive abilities (e.g. intelligence or working memory) and demographic factors (e.g. gender, education level). Although the specific factors assessed differed across studies, there was no obvious difference in the covariates controlled for in studies that did and did not yield significant partial correlations between musical ability and second-language outcomes, suggesting that observed correlations do not reflect a single confounding/underlying factor.

Nevertheless, the covariates listed above are only a subset of the many factors that might confound music–language relationships. For instance, socioeconomic status (SES) was not controlled for across any of these studies, yet SES is known to predict both participation and continued involvement in musical activities (e.g. [97–100]) and success in second-language learning [101]. Personality factors are also associated with both musical involvement [98] and second-language learning [102]. Continued research assessing the contribution of these and other potentially confounding factors is clearly an important enterprise (with the caveat that statistical control brings its own set of complications [84,85]).

Aside from our primary conclusions, this meta-analysis suggests that the literature on music and second-language learning suffers from publication bias (the unfortunately common ‘file-drawer problem’; e.g. [103]). This could result from researchers not submitting or reporting null/negative findings, the use of various questionable research practices and/or the difficulty of publishing null results. Of course, any of these reasons are problematic for our understanding of music–language relationships. While the current analysis suggests that publication bias inflates (rather than creates) the overall effect observed here, these statistical corrections are far from perfect (e.g. [104]) thus, this finding underscores a need for large-scale, pre-registered replications.

In addition to publication bias, there are several other limitations across the current literature: a majority of these studies assessed college-aged adults’ ability to learn an unfamiliar language. Of these effects, an overwhelming portion of effect sizes (80%) assessed the effect of musical ability/training on phonology-based outcomes, and 65% of the studies assessed perceptual abilities within the second language. This over-representation of certain study designs calls for more diverse approaches—specifically, the assessment of second-language syntax, semantics, and production—and a need to investigate more diverse participant populations. (By contrast, the literature has used a highly diverse set of tasks and measures to assess the same underlying constructs, which poses difficulties for cross-study comparisons [105].)

### 5.1. Conclusions and future directions

Learning new languages is becoming increasingly important as the world becomes more interconnected; as such, factors contributing to successful second-language acquisition remain important to investigate. The sum of current evidence suggests a small, but reliable, relationship between musical ability and second-language learning, with the important caveat that this literature also appears to suffer from publication bias. These relationships probably reflect some degree of shared perceptual processing [19,20] and/or shared cognitive processing (such as auditory attention [106,107] that facilitates learning of language sounds). Thus, a clearer understanding of the mechanisms that connect music and (second) language could be gained from work systematically assessing different aspects of auditory processing (e.g. spectral processing and fundamental frequency) in music and second-language learning (e.g. [108]) and assessing relative cognitive skills for processing sounds (e.g. auditory attention). In any case, it appears that musical abilities indeed predict, at least to some extent, success in second-language learning, making this a potentially fruitful area for further exploring music–language relationships.

## Data Availability

This data and analysis scripts are available on our Open Science Framework project page [109].

## Footnotes

^1^
By contrast, there is some evidence that adults can successfully learn new musical systems (e.g. [4,5]), but there has been relatively little work on this topic.

^2^
We defined musical ability as some aspect of skill in perception or production of musical notes, melodies, tones or rhythms, as measured by a test. We defined musical training as a measure of musical experience as a self-report measure. This could be treated as a continuous or a dichotomous measure.

^3^
In a separate, supplementary analysis, we treated intervention studies as musical training studies because it is a similar experience-based measurement and performed this moderator comparison. This yielded similar effects, the relationship between musical ability and second-language learning did not vary across experienced-based measurements (*z* = 0.35) and musical ability measurements (*z* = 0.30)

^4^
It should be noted that the inclusion of both bivariate and partial correlations in meta-analyses may be appropriate in some circumstances (see, e.g. [76]), however, the heterogeneity of covariates in this literature suggest that this is not such a case.
